# Comparison of Inoculation Routes for Honeybee Gut Microbiota Assembly Under Laboratory Conditions

**DOI:** 10.3390/microorganisms14081785

**Published:** 2026-08-13

**Authors:** Ming Zheng, Jiachen Xu, Jinmeng Ma, Yi Zhang, Zheguang Lin, Ting Ji, Kang Wang

**Affiliations:** College of Animal Science and Technology, Yangzhou University, Yangzhou 225009, China; zme1111@163.com (M.Z.); 15261587660@163.com (J.X.); m19505501535@163.com (J.M.); zy162207123@163.com (Y.Z.); z.lin@yzu.edu.cn (Z.L.); tji@yzu.edu.cn (T.J.)

**Keywords:** honeybees, gut microbiota, inoculation methods, microbial transmission, community assembly

## Abstract

The honeybee (*Apis mellifera*) gut microbiota serves as a model for host–microbe studies, as newly emerged workers provide a low-complexity, nearly germ-free system. Although multiple inoculation strategies are commonly applied under laboratory conditions, their comparative effects on microbial colonization remain incompletely understood. Here, we systematically compared four commonly used inoculation strategies, including pollen mixture feeding, oral feeding, dunk inoculation, and comb-exposed inoculation, under controlled laboratory conditions using an identical microbial source. We assessed gut microbiota establishment using spike-in 16S rRNA gene sequencing, enabling the quantification of both relative and absolute bacterial abundances, together with analyses of community composition, alpha diversity, and beta diversity. Our results showed that method-dependent differences were observed. Pollen mixture feeding and oral feeding produced highly comparable outcomes across the measured parameters, suggesting that dietary incorporation and direct oral delivery are similarly effective for establishing core gut communities (*p* > 0.05). Dunk inoculation also supported core bacterial colonization, although differences in beta diversity indicated some variation in community structure (*p* < 0.05). Comb-exposed inoculation showed the most pronounced differences, with significantly reduced bacterial abundance, lower alpha diversity, and decreased colonization of specific core taxa including *Bifidobacterium* and *Snodgrassella alvi* (*p* < 0.05). Importantly, despite these differences, none of the inoculation methods resulted in atypical communities dominated by noncore taxa, indicating that the core gut microbiota remained broadly conserved across treatments. Collectively, our findings indicate that although inoculation delivery methods differed fundamentally in inoculum volume, microbial concentration, and the mechanisms by which microbes were introduced into the bee gut, these distinct strategies can introduce measurable differences, while the established gut communities remain consistently dominated by core bacterial taxa. These results provide a basis for selecting appropriate inoculation strategies in experimental studies of honeybee microbiota.

## 1. Introduction

Honeybees (*Apis mellifera*) harbor a simple yet highly specialized gut microbiota composed of a limited number of dominant bacterial taxa, primarily including *Lactobacillus* Firm-5, *Bombilactobacillus* Firm-4, *Bifidobacterium*, *Snodgrassella alvi* and *Gilliamella* [1,2]. Despite its low taxonomic diversity, this microbial community performs essential functions in host nutrition, carbohydrate metabolism, immune modulation, pathogen resistance, and detoxification processes, thereby playing a fundamental role in honeybee health and colony productivity [3,4,5]. Due to its relatively stable and conserved composition, the honeybee gut microbiota has become a well-established model system for studying host–microbe interactions and microbial community assembly in social insects [6,7].

Newly emerged honeybee workers are generally considered nearly germ-free because they have not yet acquired the characteristic adult gut microbiota [8]. During pupation, renewal of the gut lining and other developmental barriers likely prevent stable transfer of larval microorganisms into the adult gut [9,10]. Accordingly, workers emerging outside the colony, or without exposure to adult bees and hive materials, usually harbor very few bacteria and may remain poorly colonized. After emergence, young workers progressively acquire a conserved core microbiota during the first week of adult life through colony-associated transmission routes [11,12,13,14]. Previous studies have demonstrated how newly emerged workers acquire their characteristic gut communities and that colonization of core symbionts depends strongly on exposure to nurse bees, hindgut-associated microbial material, and fecal transplants [11,15]. In contrast, oral trophallaxis alone and exposure to hive components such as comb, honey, and bee bread were insufficient to reliably establish a complete core microbiota [11]. More recent work has challenged this view by showing that gut microbiota assembly in honeybees is influenced by both diet and social environment [16]. Social interaction with older workers is not strictly required for core hindgut bacterial transmission under certain conditions. Instead, exposure to natural eclosion environments and fresh pollen alone can support the establishment of gut communities comparable to those of colony-reared workers. This highlights the diversity of inoculation strategies used to establish the honeybee gut microbiota.

The ability to create controlled, near-germ-free conditions and perform defined microbial inoculations makes newly emerged honeybee workers a robust model system for studying host–microbe interactions [6,17]. Currently, commonly used inoculation strategies under laboratory conditions include pollen mixture feeding, oral feeding, dunk inoculation, and comb-exposed inoculation. Pollen mixture feeding provides a diet-based and continuous exposure to microbial communities, closely mimicking natural feeding behavior; however, the exact microbial dose received by individual bees may vary, leading to reduced control over exposure consistency. Oral feeding allows precise delivery of a defined microbial dose to each individual bee, ensuring high experimental control and reproducibility, but the use of sucrose solution for individual feeding may potentially reduce microbial viability [18]. Dunk inoculation, although less commonly used, enables rapid and uniform coating of multiple individuals with microbial suspension, facilitating group-level processing; nevertheless, it may introduce variability due to handling stress and potential secondary microbial transfer during grooming interactions. Comb-exposed inoculation most closely resembles natural hive conditions by providing environmental and social contact with hive materials; however, microbial exposure in this system is indirect and difficult to quantify, which may result in lower reproducibility and higher experimental variability. However, a direct comparison of these commonly used inoculation strategies under controlled laboratory conditions is still lacking, particularly with respect to whether direct and indirect exposure routes are functionally equivalent in shaping gut microbiota assembly.

Here, we systematically compared four commonly used inoculation strategies, including pollen mixture feeding, oral feeding, dunk inoculation, and comb-exposed inoculation, using the same microbial source under controlled laboratory conditions. We evaluated whether these approaches produced consistent or divergent patterns of gut microbiota establishment, diversity, and community composition in newly emerged honeybee workers. This study provides practical guidance for establishing standardized experimental systems for honeybee gut microbiota research.

## 2. Materials and Methods

### 2.1. Rearing of Germ-Free Bees

Three healthy colonies of *Apis mellifera* were maintained in the experimental apiary of Yangzhou University (Yangzhou, China). Newly emerged workers are generally considered microbiota-free or nearly germ-free because they have not yet established the characteristic gut microbiota. To obtain a synchronized cohort, the queen of each colony was confined to a single comb frame 18 days before the experiment to allow intensive oviposition within a short period. Brood frames were then transferred to the laboratory and incubated under sterile conditions at 35 °C and 90% relative humidity until adult emergence. Dark-eyed, late-stage pupae were aseptically removed from capped brood cells and individually placed into sterile 24-well tissue culture plates for emergence. Newly emerged active workers were collected within 24 h after emergence, randomly allocated to sterile rearing cages at 20 bees per cage, with three replicate cages established for each treatment.

### 2.2. Gut Microbiota Inoculation Procedures

The inoculum was prepared from donor bees collected from the three colonies described above. Briefly, three nurse bees with visibly full guts were selected from each colony. After aseptic dissection, the guts were pooled and mechanically homogenized with a sterile pestle. The homogenate was then suspended in 800 μL of sterile PBS, repeatedly ground along the tube wall to obtain a uniform mixture, and briefly centrifuged at low speed for 15 s to sediment large debris. The resulting supernatant was used as the microbial inoculum.

Based on commonly used inoculation methods for honeybee gut bacteria, four experimental groups were established: pollen mixture (PM), oral feeding (OF), dunk inoculation (DI), and comb-exposed inoculation (CE). Throughout the experiment, all bees were provided with sterile 50% sucrose solution and pollen patties prepared from Co-60-irradiated pollen. In the PM group, the inoculum was incorporated into the pollen patty at a proportion of 100 μL microbial suspension per 1 g diet. In the other treatment groups, the same volume of sterile PBS was added to the pollen patty to ensure consistent diet moisture across groups. In the OF group, each bee was individually fed 5 μL of a 1:1 mixture of gut homogenate and sterile 50% sucrose solution. For the DI treatment, 1 mL of a 1:1 mixture of inoculum and sterile 50% sucrose solution was transferred into a sterile 50 mL Falcon tube, and 20 bees were introduced into the tube. The tube was then gently shaken for approximately 10 s to coat the bees with the inoculum suspension. After treatment, the bees were transferred to sterile rearing cages, where inoculation occurred through self- and allo-grooming. For the CE treatment, three combs were collected from each donor colony used for gut homogenate preparation and placed into a cage (50 × 50 × 50 cm). The combs contained naturally stored honey and bee bread. Newly emerged germ-free bees were introduced into the cage and exposed to the comb environment for 24 h, after which they were removed and transferred to sterile rearing cages for subsequent experiments.

All treatment groups were housed in sterile plastic cages and kept in a climate-controlled incubator at 30 °C and 60% relative humidity in continuous darkness to mimic hive conditions. Dead individuals were removed every day to minimize potential contamination. On day 8, bees were anesthetized with CO_2_, surface-sterilized in 75% ethanol, and rinsed three times with sterile distilled water. Whole guts were then aseptically dissected, immediately flash-frozen in liquid nitrogen, and stored at −80 °C until subsequent analyses.

### 2.3. Gut Microbiota Characterization

For gut microbiota characterization, the guts of seven bees were randomly sampled from each biological replicate, resulting in 15 gut samples per treatment. Genomic DNA was extracted from gut samples using the FastDNA SPIN Kit (MP Biomedicals, Santa Ana, CA, USA), which integrates mechanical disruption and chemical lysis for efficient DNA recovery.

Genomic DNA quality was evaluated by agarose gel electrophoresis and Qubit fluorometry, which were used to assess DNA integrity and concentration, respectively. Gut microbiota profiling with absolute quantification was carried out using a sequencing approach based on a spike-in internal standard method. In this system, synthetic spike-in sequences were constructed to mimic bacterial 16S rRNA genes by preserving the conserved primer-binding regions while replacing the variable regions with random sequences of approximately 40% GC content. These artificial sequences do not match any known nucleotide entries in public databases. To generate an absolute standard curve, nine spike-in standards spanning a concentration gradient selected from preliminary testing were added to each DNA sample before PCR amplification. The V3–V4 region of bacterial 16S rRNA genes, together with the spike-in standards, was co-amplified using primers 341F and 805R. Amplicons were purified using AMPure XP beads, quantified with a Qubit fluorometer, and combined at equimolar concentrations to prepare barcoded sequencing libraries according to the standard Illumina workflow. Libraries were then sequenced on the Illumina NovaSeq 6000 platform with 2 × 250 bp paired-end reads.

Raw sequencing data were analyzed in QIIME2 [19]. Primer and adapter sequences were first removed using Cutadapt (https://cutadapt.readthedocs.io/en/stable/), after which reads were quality-filtered, denoised, checked for chimeras, and inferred as amplicon sequence variants (ASVs) with DADA2 [20]. Representative ASVs were assigned taxonomic identities using a naive Bayes classifier trained on a honey bee gut microbiota reference database [21], with the confidence threshold set at 80%. Relative abundances of bacterial taxa were generated from the ASV table, and corresponding absolute abundances were estimated by normalizing these values to the total 16S rRNA gene copy numbers derived from the spike-in standards. Differences in microbial community composition among treatments were assessed using unweighted and weighted distance metrics, visualized by non-metric multidimensional scaling (NMDS), and evaluated statistically with PERMANOVA. Alpha diversity, including Shannon, Simpson, and Pielou’s evenness indices, was also calculated to compare within-sample diversity among groups.

### 2.4. Statistical Analyses

Statistical analyses were performed in R (version 4.5.1). To examine differences in bacterial abundance among inoculation treatments while accounting for the non-independence of bees housed within the same cage, we used linear mixed-effects models (LMMs), with inoculation treatment as a fixed effect and cage identity as a random effect: lmer (Response~Treatment + (1|Cage)). Absolute bacterial abundance data were log10-transformed prior to analysis, whereas relative abundance data were analyzed without transformation. Overall treatment effects were examined via ANOVA. Pairwise comparisons among treatments were performed using estimated marginal means (emmeans) with Tukey adjustment for multiple comparisons. Statistical significance was set at *p* < 0.05.

## 3. Results

### 3.1. Effects of Different Inoculation Methods on Gut Bacterial Colonization and Community Composition

**Figure 1:**
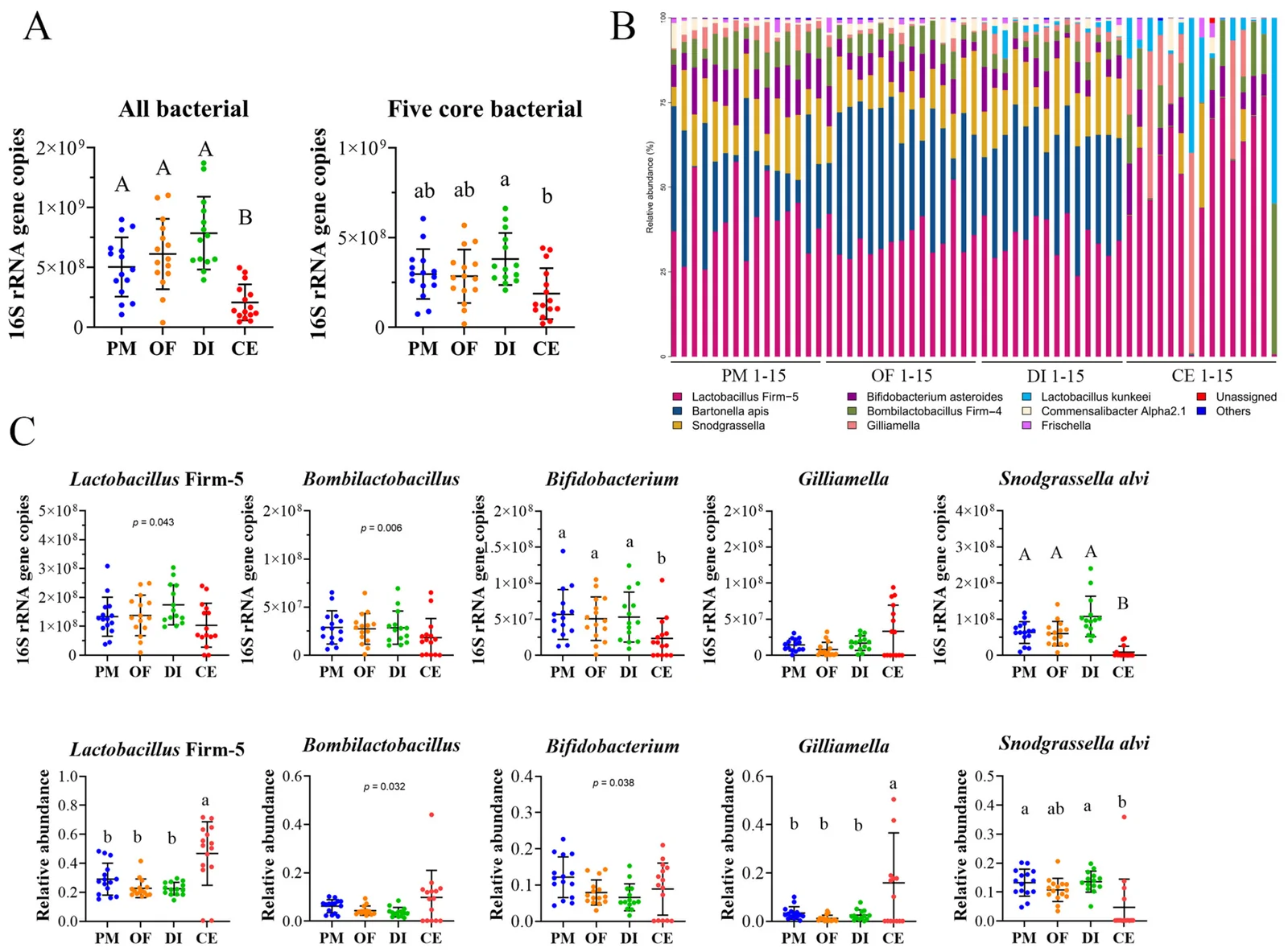
Bacterial communities established under different inoculation methods. (**A**) Absolute abundances of total bacteria and the five core gut bacteria in bees subjected to different inoculation methods. (**B**) Relative abundances of gut microbial communities in individual bees from each group. (**C**) Absolute and relative abundances of the five core gut bacteria across different inoculation methods. Uppercase letters indicate Tukey-adjusted *p* < 0.01, and lowercase letters indicate Tukey-adjusted *p* < 0.05. Exact *p* values are shown for significant overall treatment effects when no individual pairwise comparison remained significant after Tukey adjustment.

Absolute quantification of 16S rRNA gene copies revealed significant differences in total bacterial load among treatments (Figure 1A; LMM, F = 9.60, d.f. = 3, 8.16, *p* = 0.0047). Bees in the PM, OF, and DI groups exhibited comparable bacterial loads (Tukey-adjusted *p* > 0.05), whereas the CE treatment resulted in significantly lower bacterial abundance (Tukey-adjusted *p* < 0.05). A similar pattern was observed for the combined abundance of the five core bacterial taxa. No significant differences were detected among the PM, OF, and DI groups, while the CE group showed significantly reduced five core bacterial abundance (Figure 1A, Tukey-adjusted *p* < 0.05). Community composition analysis showed that the gut microbiota in the PM, OF, and DI groups exhibited broadly similar taxonomic profiles dominated by typical honeybee core bacteria (Figure 1B). In contrast, the CE treatment displayed a noticeably distinct community structure and greater inter-individual variability relative to the other inoculation methods.

Further examination of individual taxa revealed differential colonization patterns among treatments (Figure 1C). The absolute abundance of *Bifidobacterium asteroides* was significantly lower in the CE group than in the PM, OF, and DI groups (Tukey-adjusted *p* < 0.05). Similarly, *Snodgrassella alvi* showed markedly reduced colonization in the CE group (Tukey-adjusted *p* < 0.001). Although significant overall treatment effects were detected for Lactobacillus Firm-4 and Lactobacillus Firm-5, no individual pairwise comparisons remained significant after Tukey adjustment. No significant treatment effect was detected for *Gilliamella*.

Analysis of relative abundance revealed additional differences in community composition (Figure 1C). The relative abundance of Lactobacillus Firm-5 and *Gilliamella* was significantly higher in the CE group than in the PM, OF, and DI groups (Tukey-adjusted *p* < 0.05). Significant overall treatment effects were also detected for Lactobacillus Firm-4 and *B. asteroides* (*p* = 0.032 and 0.038, respectively), although no pairwise comparisons remained significant after Tukey adjustment. The relative abundance of *S. alvi* also differed significantly among treatments (*p* = 0.024), with lower abundance in the CE group than in the PM and DI groups (Tukey-adjusted *p* < 0.05), whereas the difference between CE and OF was not significant.

### 3.2. Effects of Different Inoculation Methods on Gut Microbial Diversity and Community Structure

**Figure 2:**
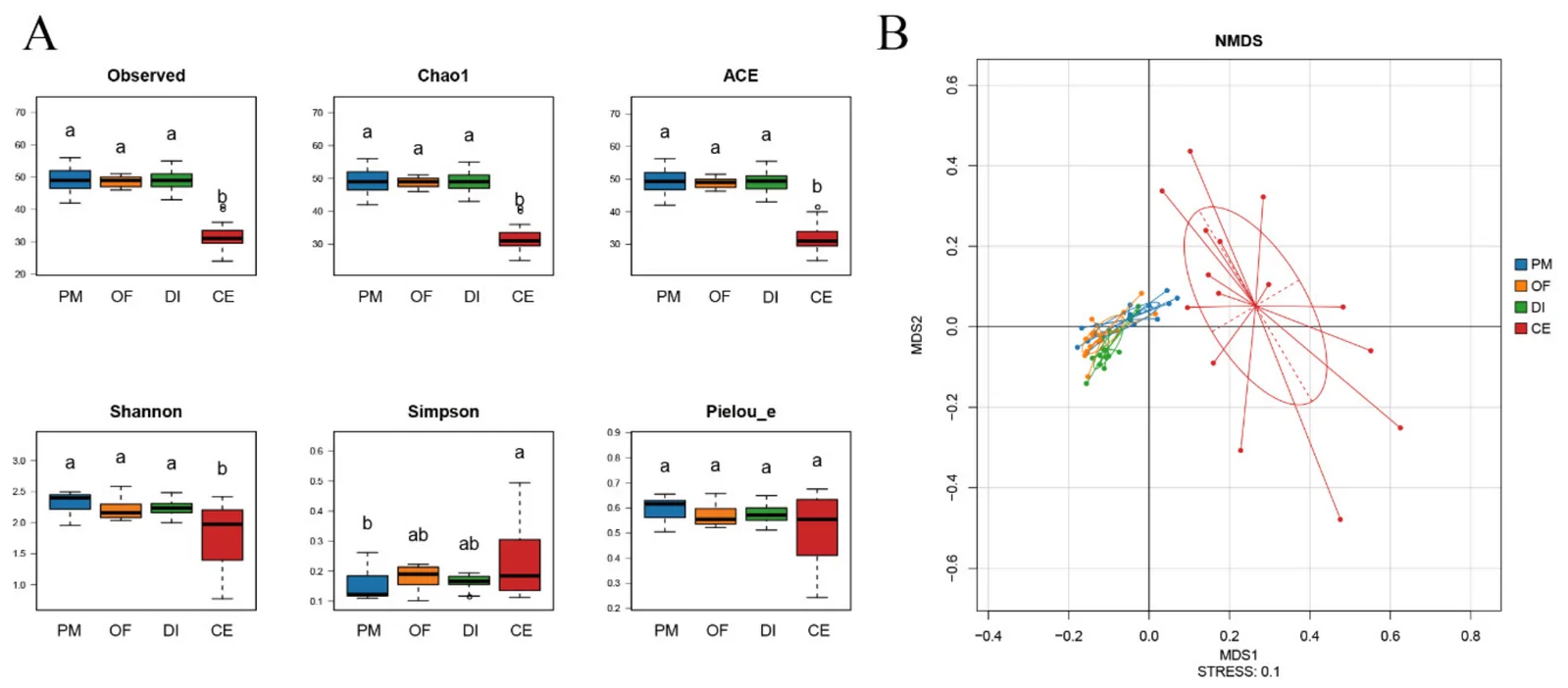
(**A**) Alpha diversity indices of gut microbial communities under different inoculation methods. (**B**) NMDS ordination based on Bray–Curtis distances showing differences in community structure among groups. Different lowercase letters indicate significant differences among groups (*p* < 0.05).

To further assess whether different inoculation methods affected community diversity and overall structure, we next compared alpha and beta diversity among groups. Alpha diversity analysis revealed that the CE group exhibited significantly lower Observed species, Chao1, ACE, and Shannon indices than the PM, OF, and DI groups (Figure 2A), while no significant differences were detected among the latter three groups. In contrast, the Simpson index was significantly increased in the CE group, suggesting that its gut microbial community was dominated by fewer taxa. Consistent with these results, NMDS analysis showed that samples from the PM, OF, and DI groups clustered closely, whereas CE samples were clearly separated and more dispersed (Figure 2B), indicating that the CE method caused a marked shift in community structure and reduced community stability. PERMANOVA analysis based on Bray–Curtis distances confirmed that these differences were statistically significant (R^2^ = 0.39, *p* < 0.001).

To better understand the group-wise effects, comparisons were evaluated across both presence/absence (unweighted) and abundance-weighted (weighted) beta diversity metrics (Table 1). Notably, Pairwise PERMANOVA further showed that PM and OF did not differ significantly in community structure under either unweighted or weighted UniFrac distances, whereas most other pairwise comparisons were significant, particularly those involving CE. This result indicates that PM and OF generated highly similar gut microbial communities, while CE produced the most distinct community structure.

## 4. Discussion

When interpreted in the context of microbial exposure route, the four inoculation strategies used here can be viewed as two broad types: direct exposure-based approaches and indirect comb-mediated exposure. Pollen mixture feeding, oral feeding, and dunk inoculation all provided relatively direct access to the same gut-derived microbial source and generally supported the establishment of core gut bacterial taxa. Among these approaches, pollen mixture feeding and oral feeding produced the most consistent outcomes, whereas dunk inoculation showed minor differences in community structure but still maintained effective core bacterial colonization. In contrast, comb-exposed inoculation, as an indirect exposure route, produced a more divergent colonization pattern. Importantly, even under comb-exposed inoculation, the community was not replaced by atypical noncore bacteria, indicating that the honeybee gut environment still favored the establishment of a core microbiota framework [22].

A more detailed comparison among inoculation methods revealed that pollen mixture feeding and oral feeding produced highly consistent outcomes across all measured parameters, indicating a strong functional equivalence between dietary incorporation and direct oral delivery. Both approaches resulted in highly similar colonization of core gut symbionts, as well as comparable patterns of microbial diversity and relative abundance, suggesting that once microbial exposure is sufficient and direct, the establishment of the dominant core microbiota is relatively robust to variation in delivery format. Although hyperosmotic sucrose solutions have been reported to reduce bacterial viability [23,24,25], oral feeding had limited effects on bacterial load and community establishment in our study. This may be because the sucrose concentration used here was relatively moderate, the inoculum was mixed with PBS as a buffering solution, and the exposure period before ingestion was short, as inoculation was completed within a few hours. These factors may have reduced osmotic stress on bacterial cells and helped maintain microbial viability during the feeding procedure. Although oral feeding allows more precise control of the inoculum dose delivered to each bee [26], it did not produce outcomes distinct from pollen mixture feeding in our study. This suggests that, when sufficient microbial exposure is provided, repeated dietary intake through pollen mixture feeding can establish the gut microbiota as effectively as direct oral administration. In addition, although pollen mixture feeding may raise concerns about microbial growth in the pollen diet and subsequent introduction into the bee gut, the comparable outcomes between pollen mixture feeding and oral feeding suggest that this effect was limited under our experimental conditions. Given that individual oral feeding is labor-intensive and time-consuming, while showing no clear advantage over pollen mixture feeding in colonization outcomes, pollen mixture feeding may be a more practical option for routine laboratory inoculation.

The subtle differences observed in the dunk inoculation group may be partially explained by the introduction of additional microbial sources during the inoculation process [27,28,29]. Although newly emerged bees are widely used as a proxy for germ-free individuals, they are more accurately described as nearly germ-free, as trace environmental or surface-associated microorganisms may still be present [30,31]. However, the absence of marked changes in alpha diversity or bacterial abundance suggests that any background microbial input introduced during dunk inoculation had a limited effect on overall core microbiota establishment. The observed beta diversity separation may therefore reflect subtle changes in community structure rather than a disruption of core bacterial colonization. This further suggests that, under direct exposure-based approaches, honeybee gut microbiota assembly tends to converge toward a conserved set of core symbionts. This pattern may partly explain why a limited number of core gut bacteria remain dominant in honeybees across different geographic regions [1,32,33]. Nevertheless, the mechanisms by which host gut environments selectively maintain these core symbionts require further investigation [34,35].

Mechanisms underlying the reduced colonization efficiency in comb-exposed inoculation may be associated with its indirect transmission pathway. Unlike pollen mixture feeding, oral feeding, and dunk inoculation, which provide direct and concentrated exposure to gut-derived microbial communities, comb-exposed inoculation relies on environmental acquisition of microorganisms from hive substrates. This indirect exposure likely reduces the effective microbial dose reaching the gut and increases stochasticity during colonization, thereby limiting the establishment efficiency of certain core symbionts. In addition, comb-associated materials such as honey and bee bread may introduce environmental microbial communities and bioactive compounds that further influence bacterial survival and colonization dynamics [36,37,38]. These factors together may contribute to the reduced abundance of key gut symbionts and the altered community structure observed in the comb-exposed group. Although previous studies have reported reductions in *Gilliamella apicola* under similar conditions [16], our results instead show decreases in *Snodgrassella alvi* and *Bifidobacterium*, suggesting that the specific taxa affected may vary depending on experimental conditions. Importantly, comb-exposed inoculation still supported the establishment of core gut bacteria, even in the absence of social contact with adult bees or direct exposure to gut homogenates. This finding is consistent with previous work showing that social interaction is not strictly required for core microbiota acquisition, and that hive-associated substrates can serve as microbial sources for newly emerged workers [16]. Therefore, although comb-exposed inoculation may partially constrain the colonization of specific core symbionts, the overall ecological niche of the honeybee gut core microbiota remains largely preserved. This suggests that, despite reduced transmission efficiency, the honeybee gut environment continues to favor the establishment of a conserved framework of core microbial taxa, preventing fundamental disruption of community structure. This finding has broader methodological implications. Before the functional importance of the honeybee gut microbiota was widely recognized, many laboratory studies used newly emerged workers collected from brood frames incubated outside the hive, thereby restricting them to comb-exposed inoculation as the sole route of microbial exposure and often treating these bees as a relatively uniform physiological starting point. Previous work showed that exposure only to hive components could lead to atypical communities dominated by noncore taxa or highly skewed bacterial compositions [11]. These observations suggest that experimental outcomes obtained from newly emerged workers may be influenced by unrecognized variation in early microbiota acquisition. However, our results, together with recent evidence showing that hive-associated substrates can support the establishment of core-dominated gut communities [16], indicate that contact with hive materials does not necessarily result in atypical microbiota. Instead, such exposure may still support the assembly of a core-dominated gut community, although with altered colonization efficiency for specific symbionts. Therefore, rather than concerns about inevitable atypical microbiota formation following comb exposure, our findings suggest that concerns about the potential impact of atypical microbiota on experimental outcomes may be unwarranted.

A limitation of this study is that taxon-specific analyses were mainly restricted to five major core bacterial taxa. These taxa were selected because they showed high detection rates and dominant abundance in our samples and represent the core bacterial members most frequently analyzed and compared in previous honeybee gut microbiota studies [1,39]. This focus enabled us to evaluate the establishment of the characteristic core community across inoculation methods in a manner consistent with the existing literature. However, this approach may have overlooked responses of low-abundance, transient, or less consistently detected bacteria. Therefore, the consistency observed among inoculation methods should be interpreted primarily with respect to the dominant core microbiota analyzed in this study. Future studies should expand to include a broader range of microbial taxa, and similar efforts are also needed in other studies to better understand the full complexity of gut microbiota.

## 5. Conclusions

This study provides a systematic comparison of different microbial inoculation strategies for establishing the honeybee gut microbiota under laboratory conditions. Our results demonstrate that the route of microbial delivery affects colonization outcomes, with direct microbial exposure approaches generally supporting more consistent establishment of core gut symbionts than indirect exposure through hive-associated materials. Nevertheless, although inoculation delivery methods differed substantially in inoculum volume, microbial concentration, and the mechanisms by which microbes were introduced into the bee gut, all tested strategies supported the formation of a gut community dominated by characteristic core bacterial taxa. These findings suggest that while inoculation procedures can influence the efficiency and fine-scale structure of microbiota assembly, the honeybee gut environment imposes strong ecological constraints that favor the establishment of a conserved core microbiota. This study provides a methodological reference for designing and interpreting experimental studies of honeybee gut microbiota.

## Figures and Tables

**Table 1:** Pairwise PERMANOVA among inoculation groups.

Comparison	Unweighted UniFrac			Weighted UniFrac		
	F. Model	R^2^	Pr (>F)	F. Model	R^2^	Pr (>F)
PM vs. OF	1.2106	0.0429	0.2747	1.6638	0.0581	0.0599
PM vs. DI	4.6809	0.1478	0.0284	3.6054	0.1178	0.0019
PM vs. CE	14.7889	0.3456	0.0001	4.8314	0.1472	0.0013
OF vs. DI	4.3141	0.1336	0.0347	2.5767	0.0843	0.0244
OF vs. CE	31.1577	0.5267	0.0001	4.3963	0.1354	0.0024
DI vs. CE	31.3712	0.5374	0.0001	6.5553	0.1954	0.0001

## Data Availability

Raw sequencing data are publicly available in the Genome Sequence Archive at the China National Genomics Data Center under accession number CRA045554.

## Abbreviations

PM	Pollen mixture
OF	Oral feeding
DI	Dunk inoculation
CE	Comb-exposed inoculation
